# Physiological Responses of Two Olive Cultivars to Salt Stress

**DOI:** 10.3390/plants12101926

**Published:** 2023-05-09

**Authors:** Olfa Boussadia, Hatem Zgallai, Nada Mzid, Rihem Zaabar, Mohamed Braham, Georgios Doupis, Georgios Koubouris

**Affiliations:** 1 Olive Institute, Ibn Khaldoun BP 14, Sousse 4061, Tunisia; 2 National Institute of Agronomic Research of Tunisia, Rue Hedi Karray, Tunis 1004, Tunisia; 3 Department of Agriculture Forestry and Nature (DAFNE), University of Tuscia, 01100 Viterbo, Italy; 4 Laboratory of Olive Cultivation, Institute of Olive Tree, Subtropical Crops and Viticulture, Hellenic Agricultural Organization DIMITRA, Leoforos Karamanli 167, 73134 Chania, Crete, Greece

**Keywords:** antioxidant enzymes, climate change, K^+^/Na^+^ ratio, *Olea europaea* L., photosynthesis, plant phenotyping

## Abstract

The olive tree (*Olea europaea* L.) is the main fruit tree in most of the arid and semi-arid regions of Tunisia, which is where the problem of salinity is more pronounced. Salinity is one of the main factors that affects the productivity of olive trees, so the objective of this experiment was to study the effects of salinity on the photosynthesis, water relations, mineral status, and enzymatic activity of two cultivars of *Olea europaea* L., ‘Chemlali’ and ‘Koroneiki’. The trial was conducted under controlled conditions in a greenhouse for a period of 49 days and included two treatments: T0 control and T100 (irrigation with 100 mM of NaCl solution). Under salinity stress, the photosynthesis, stomatal conductance, and leaves of both cultivars were negatively affected. ‘Chemlali’ showed greater tolerance to NaCl salinity, based on a progressive decrease in osmotic potential (Ψπ) followed by a progressive and synchronous decrease in gs, without a comparable decrease in photosynthesis. The water use efficiency (WUE) improved as a result. In addition, the K^+^/Na^+^ ratio in ‘Chemlali’ rose. This appears to be crucial for managing stress. Conversely, enzymatic activity showed an accumulation of glutathione peroxidase (GPX) in stressed plants. The catalase (CAT) and ascorbate peroxidase (APX) content decreased in both stressed varieties. It can be concluded that the cultivar ‘Koroneiki’ is more susceptible to salt stress than the cultivar ‘Chemlali’, because the accumulation of GPX and the decreases in CAT and APX were more pronounced in this cultivar.

## 1. Introduction

Numerous environmental stresses are caused by climate change. Salinity is the second most important abiotic factor affecting agricultural productivity worldwide. It interferes with numerous physiological, biochemical, and molecular processes [1]. The use of low-quality water (brackish water, reclaimed water, drainage), often with high salinity levels, is becoming increasingly important in order to overcome water scarcity and to meet the growing demand for water for agricultural development [2,3,4,5]. Olives, one of the most important tree crops in the Mediterranean, are often exposed to high salinity levels in the root zone, especially during long, hot, and dry summers [2,6,7]. Olive is a moderately salinity-tolerant species, with significant differences in salt tolerance between cultivars [3].

In order to provide insights and solutions for improving yield and water use efficiency (WUE), a better understanding of the physiological mechanisms underlying crop growth under salinity stress is needed [8]. Salinity induces compatible solute synthesis and regulates ion migration [9]. Salt stress induces osmotic stress and ion toxicity in plants, resulting in growth inhibition, developmental modifications, metabolic limitations, and ion sequestration or exclusion [10,11]. The effects of salt stress on leaf stomatal conductance, photosynthesis, and intrinsic WUE vary depending on the degree and duration of stress, as well as on the plant species or cultivar [12]. Previous studies have shown that leaf osmotic potential [10] and leaf nitrogen content [13] are closely related to stomatal conductance and photosynthesis. Several studies have shown that osmotic adjustment is the main controlling factor of stomatal conductance in plants that are growing in saline soils [11,14,15].

Tolerance may also involve osmotic adjustments at the cellular level [16]. Some plants implement this process by increasing the amount of solutes and decreasing the water potential of the root cells, thus counteracting the efflux of water [3]. 

A balance between the osmotic potential in the vacuole and in the cytoplasm must be maintained in salt-stressed plants. The cytoplasmic accumulation of some organic solutes, such as soluble sugars and proline, helps to regulate osmotic potential from the cell to the entire plant [17,18,19,20,21,22].

Osmoregulation is important for maintaining positive turgor potential in leaves and for maintaining metabolic processes [23]. One of the mechanisms leading to the reduction of leaf water potential, and possibly leading to the reduction of potassium (K^+^) and mineral nutrient imbalances, is the accumulation of sodium (Na^+^) and chloride (Cl^−^) in leaves [2,24,25]. 

The accumulation of Na+ ions in the leaf leads to an increase in mesophyll resistance, combined with an increase in stomatal resistance, which gradually reduces the amount of CO_2_ reaching the chloroplast [21,26]. In turn, this decrease in CO_2_ concentration leads to decreased photosynthesis [22,26,27]. Photosynthesis and stomatal conductance are well correlated [22,26,28]. A decreased photosynthetic rate may also be caused by other nonstomatal limitations, including altered photosynthetic metabolism and the inhibition of Calvin Cycle enzymes, such as Rubisco [29,30].

Salt stress can contribute to the production of enormous reactive oxygen species (ROS) [3,21]. ROS are known to generate damage to cell membranes and other cellular components [31,32,33]. Excessive ROS production leads to lipid peroxidation, enzyme inhibition, and nucleic acid modifications [34,35,36]. However, plants are able to develop tolerance in adverse conditions after strong antioxidative enzyme production [3,32,37,38,39,40]. Among these enzymes, superoxide dismutase (SOD), ascorbate peroxidase (APX), and glutathione reductase (GSH) are chloroplast and mitochondrial localizations [35,41,42], whereas catalase (CAT) and guaiacol peroxidase (GPX) are generally found in microbodies and the cytosol, respectively [39,43,44]. 

In the following years, Tunisia needs to intensify olive production in order to increase the productivity of olive trees and remain competitive on the global market. Some local farmers are switching from rain-fed to irrigated and fertilized olive groves. The water used to irrigate olive groves is often saline, especially in coastal, central, and southern Tunisia. Farmers are also introducing promising foreign olive varieties into orchards and are growing them under the same irrigation and fertilization regimes as local varieties in order to maximize the yield and quality of the olives. 

In this context, it is important to select varieties that perform well when grown on saline soils or irrigated with saline water. Among the cultivars studied in this work, ‘Chemlali’ (Tunisian cultivar) and ‘Koroneiki’ (introduced Greek cultivar) are of increasing interest to Tunisian farmers. The aim of this work was to study the behavior of the two olive cultivars under salinity stress through the analysis of water relations, gas exchange parameters, mineral content, and antioxidant enzyme activity. This study will allow us to understand how to intervene through fertilization to mitigate the effects of salinity stress on the productivity of olive trees. 

## 2. Results

### 2.1. Impact of Salt Stress on the Osmotic Potential and Osmotic Adjustment of Olive Trees

**Figure 1:**
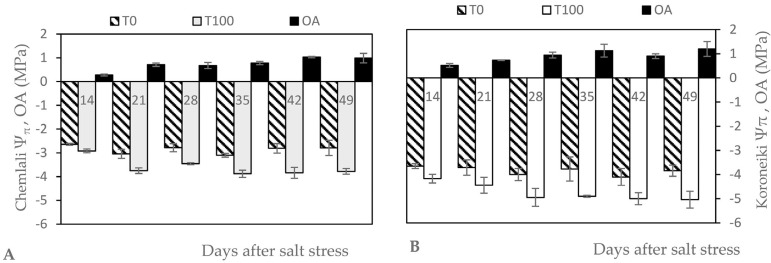
Effect of salt stress on osmotic potential (Ψπ, MPa) and osmotic adjustment (OA, MPa) of leaves of *Olea europaea* L. ‘Chemlali’ (**A**) and ‘Koroneiki’ (**B**). Each value represents the average of three measurements—Duncan (*p* = 0.05).

Figure 1 shows the progressive effects of salt stress on osmotic potential (Ψπ). Indeed, Ψπ showed a significant gradual decrease in the olive trees that were exposed to salt stress, whereas the value was rather constant in the control trees. The lowest Ψπ was reached towards the end of the experiment for ‘Koroneiki’. Compared to the control, the reduction was 26% and 24% for ‘Chemlali’ and ‘Koroneiki’, respectively. In ‘Koroneiki’, OA remained constant after 21 days of stress until the end of the experiment, with a mean value of 1.01 MP. In ‘Chemlali’, the OA increased significantly after 21 days of salt stress and again after 42 days of salt stress, with a mean value of 1 MP at the end of the experiment.

### 2.2. Impact of Salt Stress on Photosynthesis and Stomatal Conductance of Olive Plants

**Figure 2:**
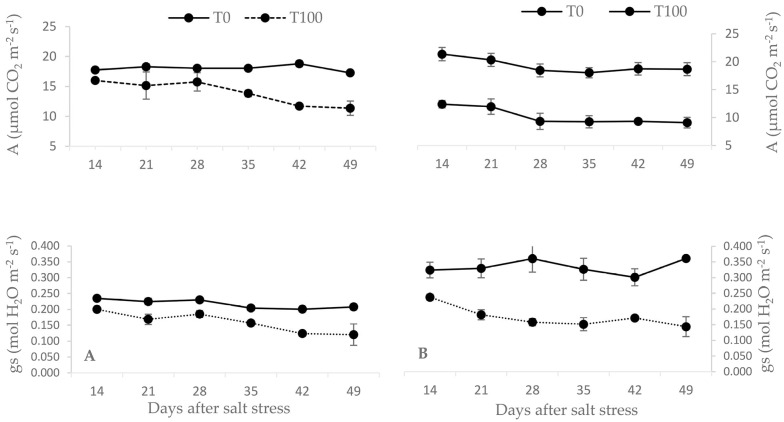
Effect of salt stress on the photosynthesis (*A*, μmol CO_2_ m^−2^ s^−1^) and stomatal conductance (*gs*, mol H_2_O m^−2^ s^−1^) of *Olea europaea* L. leaves. ‘Chemlali’ (**A**) and ‘Koroneiki’ (**B**). Each value represents the average of three measurements-Duncan (*p* = 0.05).

Figure 2 indicates how the salt stress treatment affected the photosynthetic capacity of the leaves (*A*, mol CO_2_ m^−2^ s^−1^) of the olive plants ‘Chemlali’ and ‘Koroneiki’. Salt stress treatment had a considerable impact on the photosynthetic capacity of the leaves of both cultivars after 49 days. There was a 34% decrease in ‘Chemlali’ from T0 to T100. For ‘Koroneiki’, the decrease reached 51% from T0 to T100. A significant decrease of *A* in the ‘Koroneiki’ cultivar started after 14 days of salt stress and became significantly stable after 28 days, with a mean value of 9.3 μmol CO_2_ m^−2^ s^−1^, whereas in ‘Chemlali’, a significant decrease started after 35 days of salt stress and stabilized significantly after 42 days, with a mean value of 11.55 μmol CO_2_ m^−2^ s^−1^. 

The stomatal conductance (*gs*) of both cultivars decreased as a result of salt stress treatment (Figure 2). At the end of the experiment, the control plants for ‘Chemlali’ and ‘Koroneiki’ displayed *gs* values of 0.208 and 0.361 mol H_2_O m^−2^ s^−1^, respectively. However, the gs values for the T100 salt stress plants decreased for ‘Chemlali’ and ‘Koroneiki’ by 58% and 40%, respectively. The weekly evolution of *gs* in ‘Chemlali’ showed a significant progressive decrease from 21 days to 42 days of salt stress. Compared to ‘Koroneiki’, the weekly evolution of *gs* showed a significant decrease starting after 21 days of stress and remained constant until the end of the experiment. 

### 2.3. Impact of Salt Stress on Water Use Effeciency of Olive Trees

**Figure 3:**
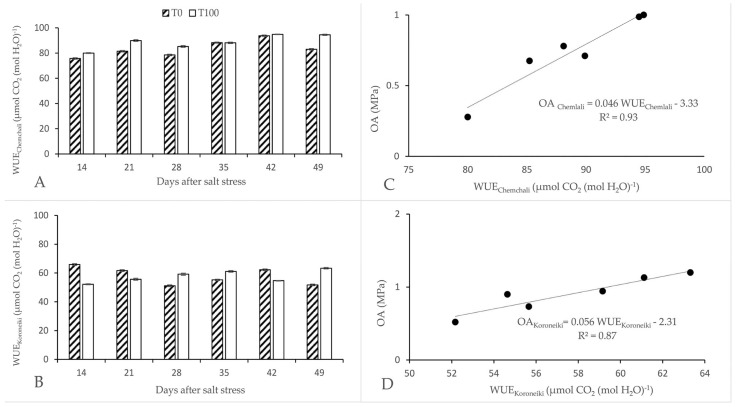
Effect of salt stress on water use efficiency (WUE, µmol CO_2_ (mol H_2_O)^−1^) of *Olea europaea* L. ‘Chemlali’ (**A**) and ‘Koroneiki’ (**B**). Each value represents the average of three measurements—Duncan (*p* = 0.05). The relationship between OA_Chemlali_ and WUE_Chemlali_ (**C**) and between OA_Koroneiki_ and WUE_Koroneiki_ (**D**). The data are fitted with regression lines. The equation for these relationships is as follows: OA _Chemlali_ = 0.046 WUE_Chemlali_-3.33 (r^2^ = 0.93; *n* = 6; *p* = 0.001); OA_Koroneiki_= 0.056 WUE_Koroneiki_-2.31 (r^2^ = 0.87; *n* = 6; *p* = 0.001).

The intrinsic WUE of ‘Koroneiki’, defined as the ratio A/gs, increased after 28 days of salt stress, but decreased sharply thereafter (Figure 3B). An increase in the WUE of ‘Chemlali’ was observed after 14 days of salt stress (Figure 3A). The WUE of ‘Chemlali’ showed a progressive increase with increasing salt stress. Compared to the control, a maximum increase of 12% was reached towards the end of the experiment. On the other hand, the efficiency of ‘Koroneiki’ was not synchronized with the progression of salt stress. The highest value of WUE was recorded towards the end of the experiment, with an increase of 20% compared to the control. The intrinsic WUE of ‘Chemlali’ and ‘Koroneiki’ was 94.49 and 63.3 μmol CO_2_/mol H_2_O, respectively, after 49 days of salt stress. A significant relationship between WUE and OA was observed for both cultivars (Figure 3C,D). The improvement in WUE was due to the maintenance of photosynthetic capacity and the decrease in stomatal conductance.

### 2.4. Impact of Salt Stress on Ion Concentration in Leaves and Roots of Olive Trees

For the ‘Chemlali’ and ‘Koroneiki’ cultivars, Na^+^ concentration in the leaves increased with salinity by 23% and 26%, respectively. In contrast, K^+^ concentration in the leaves decreased with salinity by 2% and 16% for ‘Chemlali’ and ‘Koroneiki’, respectively. In the roots of both cultivars, K^+^ concentration increased by 9% in ‘Chemlali’ and by 24% in ‘Koroneiki’ (Table 1).

The K^+^/Na^+^ ratio in the leaves was reduced by 37% and 39% for ‘Chemlali’ and ‘Koroneiki’, respectively, compared to T0 (Table 1). For the roots, the K^+^/Na^+^ ratio increased by 9% and 27% for ‘Chemlali’ and ‘Koroneiki’, respectively.

This reduction in the leaf ratio was associated with a reduced accumulation of K^+^ and a higher concentration of Na^+^ in the leaves of the plants that were treated with saline water. The increase in the root ratio can be explained by the increase in K^+^. 

### 2.5. Impact of Salt Stress on Antioxidant Enzyme Activity

At the end of the experiment, in the leaves of the plants that were treated with 100 mM of NaCl, ascorbate peroxidase (APX) and catalase (CAT) activities systematically decreased, compared to controls across the two cultivars at the end of experimentation (Table 2). CAT activities decreased by 78% and 71% for ‘Chemlali’ and ‘Koroneiki’, respectively. APX activities decreased by 44% and 52% for ‘Chemlali’ and ‘Koroneiki’, respectively. GPX increased more markedly, with increases of 28% and 29% for ‘Chemlali’ and ‘Koroneiki’, respectively.

## 3. Discussion

Ψπ is an important indicator used to express the water status of plants. The Ψπ measured for both control and stressed ‘Chemlali’ and ‘Koroneiki’ ‘trees can be used as a first indication about the trees’ response to salt stress. Both cultivars showed a gradual decrease in their potential during the course of salt stress.

The reduction in leaf Ψπ induced stomatal closure, as indicated by the decrease in *gs* (Figure 2). As a consequence, the rate of gas exchange became more and more limited. Compared to the control olive trees, photosynthesis (*A*) decreased significantly in ‘Koroneiki’ and more moderately in ‘Chemlali’ under salt stress. These results are in agreement with the findings of previous studies [3,22,45,46,47,48,49,50,51]. 

The effect of salinity on the rate of CO_2_ assimilation varied according to the concentration of salt to which the plants were exposed and according to the cultivar. In general, the highest inhibition is observed in olive cultivars with naturally high rates of photosynthesis and stomatal conductance [26], in the case of ‘Koroneiki’. Our results showed a significant decrease in the assimilation rate at the end of the experiment. This was observed for both cultivars that were exposed to 100 mM of NaCl for 49 days. This decrease varied from 34% for the salt tolerant ‘Chemlali’ to 51% for the moderately sensitive ‘Koroneiki’ (Figure 2). Compared to ‘Chemlali’, the decrease in *A* started very early in ‘Koroneiki’. This is an indication that ‘Chemlali’ activates a faster response to salt stress.

In salt stressed olive plants, changes in photosynthesis are preceded by a decrease in stomatal conductance (*gs*) [52]. Thus, the reduced CO_2_ uptake was due to hydroactive stomatal closure. Stomatal closure reduces water loss by transpiration, which leads to altered chloroplast activity as a result of altered chloroplast light harvesting and energy conversion systems [52]. ‘Chemlali’ showed a progressive decrease in *gs*, which aligned with the progression of stress. This cultivar decreased its conductance more than ‘Koroneiki’, while maintaining its photosynthesis for as long as possible. The degree of reduction in *gs* may be due to stomatal closure as a result of the excessive accumulation of Na^+^ ions in the guard cells, which reduces the availability of internal CO_2_ [53,54]. 

The photosynthetic response is always adjusted by *gs*, resulting in a change in WUE when plants are exposed to osmotic stress. The salinity effect on WUE varies with the intensity and duration of stress [55,56]. WUE was improved under a certain stress range, but this improvement was dependent on the cultivar. At the end of the experiment, the WUE of ‘Chemlali’ and ‘Koroneiki’ increased by 12% and 20%, respectively. ‘Chemlali’ exhibited a progressive improvement in WUE since the beginning of the application of salt stress. We found that salt stress reduced the osmotic potential of ‘Chemlali’ leaves, leading to a greater reduction in *gs* without compromising the photosynthetic capacity. 

An increase in leaf WUE under salt stress may be an indicator of salinity tolerance [57]. The intrinsic WUE was improved by decreasing *gs* via an increase in osmotic adjustment (Figure 3) and hydraulic resistance, and decreasing *A* via an increase in stomatal limitation, rather than a decrease in photosynthetic capacity [15].

Our results confirm the finding obtained for tomatoes grown under high salinity, which showed that WUE was significantly increased due to a decrease in *gs*, which exceeded the decrease in *A* [56]. This is an indication that salinity may have an effect on WUE. 

One important mechanism of salt tolerance in plants is their ability to store salts in their roots, thereby avoiding the transport of toxic ions to the aerial parts of the plant [58]. The lower Na^+^ concentration in the leaves of ‘Chemlali’ compared with ‘Koroneiki’ is due to the greater accumulation of this ion in the roots. This assumption is supported by previous results [47,59,60], where differences in the content of Na^+^ and Cl^−^ ions in the leaves of olive cultivars were related to more efficient mechanisms of Na^+^ and Cl^−^ retention in the roots. The higher K^+^/Na^+^ ratio of ‘Chemlali’ (Table 1) was associated with reduced Na^+^ accumulation and higher K^+^ concentrations in the roots of saline-treated plants, as compared to ‘Koroneiki’. Tattini [61] reported that the higher salinity tolerance of ‘Frantoio’ compared to the sensitive ‘Leccino’ was related to maintaining a higher K^+^/Na^+^ ratio in young leaves. Our results indicate that ‘Chemlali’ may be less sensitive to salinity because of its ability to maintain a higher K^+^ and K^+^/Na^+^ ratio than ‘Koroneiki’.

Salt accumulation in the root zone causes the development of osmotic stress (osmotic effect) and disrupts cell ion homeostasis by inhibiting the uptake of essential nutrients and inducing the accumulation of Na^+^ and Cl^−^ to potentially toxic levels within cells (specific ion effect) [62,63]. These primary stresses induce the generation of reactive oxygen species (ROS) [33,64,65], causing a reduction in the activity of certain enzymes [33,64,66]. 

The first line of defense against excess ROS is usually antioxidant enzymes [67]. The decreased stimulation of CAT and APX activities under salt stress suggest that H_2_O_2_ was utilized by GPX, reducing its presumed damaging action in ‘Chemlali’ and ‘Koroneiki’ trees (Table 2). Consistent with these findings, Regni et al. [3] showed that CAT was not significantly elevated in the ‘Koroneiki’ cultivar under salt stress.

Araújo et al. [32] also reported the upregulation of GPX activity in field-grown olive trees (cv. Cobrançosa) in response to water deficit. In turn, Sofo et al. [68] showed that APX, CAT, and GPX activities increased with increasing water deficit in young potted ‘Coratina’ olive plants. However, in olive trees, CAT, GPX, and APX enzymes are essential for the maintenance of membrane integrity [32].

To summarize, decreased photosynthesis resulting from salt stress could be related to stomatal and/or nonstomatal factors [69]. Salt stress has both direct and indirect effects on the chlorophyll content and photosynthetic efficiency of plants. Direct effects are achieved by regulating the activity and expression of the enzymes involved in chlorophyll biosynthesis and photosynthesis. Indirect effects take place through specific regulatory pathways, such as those of antioxidant enzymes [45,70].

## 4. Materials and Methods

### 4.1. Plant Material and Treatments

At the Tunisian Olive Institute (Tunisia, 35°49′ N, 10°38′ E), one-year-old olive trees (‘Chemlali’ and ‘Koroneiki’) were cultivated in 4 L plastic pots under typical daylight conditions. Before starting the experiment, trees that were about 1 m tall were selected. They were lifted from a soil mixture of organic matter, sand, and clay. The plants were then planted in a substrate mixture containing perlite and peat (1/2 volume). The trees were irrigated daily to field capacity with Hoagland’s full-strength solution for 8 weeks. 

From 6 April to 30 May 2019, the plants were subjected to salt stress. The control plants were watered daily to field capacity (saturation), whereas the salt stress treatments were imposed gradually. Two treatments were considered: 

T0: daily irrigation with nutritive solution. 

T100: daily irrigation with 100 mM of NaCl added to the nutritive solution.

The mean day/night temperatures were 32 °C and 18 °C, respectively, and the mean day/night humidity was 65% and 85%, respectively, during the salt stress experiment. The control trees and the salt-stressed trees were arranged in a completely randomized design with four replications for each cultivar and for each treatment. A total of 16 olive trees were used.

### 4.2. Osmotic Potential

The leaf osmotic potential (Ψπ) was measured on fully expanded leaves that were exposed to sunlight (taken from the middle part of the shoot). Following the method described by [71], the leaves of four plants per treatment and per cultivar were measured at midday (12 a.m.) using a thermocouple psychrometer (sample chambers type C52; Wescor, Logan, UT, USA). Over a period of 49 days, measurements were taken weekly. One leaf disc sample was taken from each plant and each measurement event leaf. In order to disrupt the cell membranes, the disc sample was wrapped in aluminum foil and placed in a freezer at −20 °C for 24 h. The disc was used to measure Ψπ after thawing.

### 4.3. Simultaneous Gas Exchange Measurements

A portable gas exchange system (LI-6400, LI-COR, Lincoln, NE, USA) was used to simultaneously measure the photosynthetic assimilation rate (*A*, μmol CO_2_ m^−2^ s^−1^) and stomatal conductance to water vapor (*gs*, mol H_2_O m^−2^ s^−1^) in the fully expanded leaves. The measurements were performed weekly over a period of 49 days in three replications for each treatment and cultivar under conditions of light saturation (1500 μmol PAR m^−2^ s^−1^) between 10 am and 2 pm and at a fixed CO_2_ concentration (Ca of 400 μmol mol^−1^), leaf temperature (25 °C), and relative humidity (60%) [22].

### 4.4. Mineral Nutrient Analysis

At the end of the experiment, the mineral composition of each plant organ (roots and leaves) was determined. The leaves, shoots, and roots were harvested and analyzed to obtain their sodium and potassium content. Deionized water was used for intensive washing of the roots. 

The sodium (Na^+^) and potassium (K^+^) contents (% DW) of the old olive leaves were determined according to the method of Martin-Prével et al. [72]. For each treatment and for each variety, eight leaves were collected from four plants, pooled into a composite sample, dried at 70 °C for 48 h, and were then crushed. One gram of this material was calcined and transferred to nitric acid. A flame photometer (Jenway, England) was used to determine the K^+^ and Na^+^ contents.

### 4.5. Antioxidant Enzyme Analysis

During R.Z.’s research visit to the Institute of Olive Tree, Subtropical Crops, and Viticulture, ELGO-DIMITRA in Greece, antioxidant enzyme analysis was carried out. With 0.1 M of phosphate buffer (pH 7), 0.5 M of Na2EDTA, 1% PVP (*m*/*v*), 1 mM of PMSF, 0.2% Triton X-100 (*v*/*v*), and 2mM of DTT, frozen olive leaf powder was homogenized. The supernatant was used to assess the activities of catalase (EC 1.11.1.6), ascorbate peroxidase (EC 1.11.1.11), and glutathione peroxidase (EC 1.11.1.7). In order to determine the CAT activity, 500 mL of ultrapure water, 0.1 mL of potassium phosphate buffer (pH 7), 50 mL of the supernatant, and 6 mM H_2_O_2_ were combined [73]. The following ingredients were combined: 500 mL of ultrapure water, 50 mL of the supernatant, and 6 mM H_2_O_2_. Five minutes after the reaction began, 1 mL of titanium reagent—1 g of titanium dioxide and 10 g of potassium sulfate dissolved in 150 mL of sulfuric acid—was added to stop it. A centrifuge was used to separate the mixture (10,000× *g*, 10 min, 4 °C), and the absorbance was measured at 415 nm. A calibration curve created with a known concentration of catalase (y = 0.4639e − 23.32x and R^2^ = 0.98) was used to measure CAT activity [73]. The Nakano and Asada method [74] was used to measure the activity of APX. The reaction mixture included supernatant, 0.1 mM of EDTA, 50 mM of potassium phosphate buffer (pH 7.5), and 0.5 mM of ascorbic acid. The reduction of ascorbate was observed at 290 nm and the reaction was started using 0.1 mM of H_2_O_2_. In order to determine the activity of APX, the molar extinction coefficient of ascorbic acid (ε = 2.8 mM^−1^ cm^−1^) was utilized. The activity of GPX was measured using the procedure outlined by Pütter [75]. By including the supernatant, 15 mM of guaiacol, and 100 mM of phosphate buffer (pH 7), GPX activity was determined. A total of 3 mM of H_2_O_2_ was used to start the reaction. GPX activity was determined using the tetraguaiacol molar extinction coefficient (ε = 26.6 mM^−1^ cm^−1^) to monitor glutathione oxidation at 470 nm.

### 4.6. Statistical Analysis

The data analysis was performed using SPSS 21.0. The significance of the changes between treatments was assessed using the two-way ANOVA model. The Duncan test [76] revealed significant differences at *p* = 0.05.

## 5. Conclusions

The irrigation of olives with salt water will inevitably increase in the Mediterranean area in the future, especially in countries with semi-arid or arid climates. This is the reason why we decided to install this experiment. Salt treatment at the level of 100 mm of NaCl caused negative effects on leaf gas exchange, ion concentration, and the enzymatic activity of olive cultivars ‘Chemlali’ and ‘Koroneiki’. Indeed, photosynthesis, stomatal conductance, and the leaves of both cultivars were negatively affected. However, ‘Chemlali’ showed greater tolerance to NaCl salinity based on a progressive decrease in Ψπ, followed by a progressive and synchronous decrease in *gs* with the application of salt stress, without a similar decrease in photosynthesis. This explains the improvement in its WUE.

In ‘Chemlali’, the K^+^/Na^+^ ratio increased under salinity stress. This seems to play an important role in tolerating stress. The accumulation of GPX and the decrease in CAT and APX were more pronounced in ‘Koroneiki’ and perhaps confirmed that this cultivar is more sensitive to salt stress than ‘Chemlali’. In order to mitigate the effects of salinity on olive trees, supplementation with potassium or amino acid fertilizers must be studied in detail.

## Figures and Tables

**Table 1:** Effect of NaCl salinity on the concentration of Na, K, and K^+^/Na^+^ in the leaves and roots of olive ‘Chemlali’ and ‘Koroneiki’ at the end of the experiment.

		Leaves			Roots		
Cultivar	T	K+ (%dw)	Na+ (%dw)	K+/Na+	K+ (%dw)	Na+ (%dw)	K+/Na+
Chemlali	T0	0.52 ± 0.02 ^A,a^	0.10 ± 0.02 ^B,a^	5.2 ± 1.11 ^A,a^	0.43 ± 0.07 ^A,a^	0.20 ± 0.04 ^A,a^	2.15 ± 0.19 ^A,a^
	T100	0.51 ± 0.02 ^A,a^	0.13 ± 0.04 ^A,b^	3.92 ± 0.03 ^A,b^	0.47 ± 0.04 ^A,a^	0.20 ± 0.04 ^A,a^	2.35 ± 0.05 ^A,b^
Koroneiki	T0	0.56 ± 0.02 ^AB,a^	0.11 ± 0.03 ^B,a^	5.09 ± 1.5 ^A,a^	0.38 ± 0.05 ^A,a^	0.24 ± 0.00 ^A,a^	1.58 ± 0.19 ^B,a^
	T100	0.47 ± 0.03 ^B,b^	0.15 ± 0.02 ^A,a^	3.13 ± 0.05 ^B,b^	0.50 ± 0.12 ^A,ab^	0.23 ± 0.03 ^A,a^	2.17 ± 0.27 ^A,b^

The data is presented as mean and standard deviation. Significant differences (*p* < 0.05) between various cultivars for the same treatment are indicated by different uppercase letters in the same column. Different lowercase letters in the same column indicate significantly different values (*p* < 0.05) between different treatments for the same cultivar.

**Table 2:** Effect of salt stress on catalase (CAT), glutathione peroxidase (GPX), and ascorbate peroxidase (APX) accumulation.

Cultivar	Treatment	CAT (Units g^−1^ FM)	GPX (Units g^−1^ FM)	APX (Units g^−1^ FM)
Chemlali	T0	170 ± 24 ^A,a^	13.8 ± 0.8 ^B,ab^	0.825 ± 0.487 ^AB,a^
	T100	38 ± 8 ^A,b^	19.2 ± 16.8 ^AB,a^	0.362 ± 0.287 ^A,b^
Koroneiki	T0	185 ± 75 ^A,a^	13.6 ± 3.2 ^B,b^	1.037 ± 0.575 ^A,a^
	T100	54 ± 2.5 ^A,b^	47.2 ± 0.8 ^A,a^	0.500 ± 0.175 ^A,b^

The data is presented as mean and standard deviation. Significant differences (*p* < 0.05) between various cultivars for the same treatment are indicated by different uppercase letters in the same column. Different lowercase letters in the same column indicate significantly different values (*p* < 0.05) between different treatments for the same cultivar.

## Data Availability

The data is contained within the manuscript.

## References

[1] Raza A., Tabassum J., Fakhar A.Z., Sharif R., Chen H., Zhang C., Ju L., Fotopoulos V., Siddique K.H.M., Singh R.K. (2022). Smart Reprograming of Plants against Salinity Stress Using Modern Biotechnological Tools. Crit. Rev. Biotechnol..

[2] Chartzoulakis K., Psarras G., Vemmos S., Loupassaki M., Bertaki M. (2006). Response of Two Olive Cultivars to Salt Stress and Potassium Supplement. J. Plant Nutr..

[3] Regni L., Del Pino A.M., Mousavi S., Palmerini C.A., Baldoni L., Mariotti R., Mairech H., Gardi T., D’Amato R., Proietti P. (2019). Behavior of Four Olive Cultivars during Salt Stress. Front. Plant Sci..

[4] Kourgialas N.N., Koubouris G.C., Dokou Z. (2019). Optimal Irrigation Planning for Addressing Current or Future Water Scarcity in Mediterranean Tree Crops. Sci. Total Environ..

[5] Mzid N., Boussadia O., Albrizio R., Stellacci A.M., Braham M., Todorovic M. (2023). Salinity Properties Retrieval from Sentinel-2 Satellite Data and Machine Learning Algorithms. Agronomy.

[6] Remorini D., Melgar J.C., Guidi L., Degl’Innocenti E., Castelli S., Traversi M.L., Massai R., Tattini M. (2009). Interaction Effects of Root-Zone Salinity and Solar Irradiance on the Physiology and Biochemistry of *Olea Europaea*. Environ. Exp. Bot..

[7] Petridis A., Therios I., Samouris G., Koundouras S., Giannakoula A. (2012). Effect of Water Deficit on Leaf Phenolic Composition, Gas Exchange, Oxidative Damage and Antioxidant Activity of Four Greek Olive (*Olea europaea* L.) Cultivars. Plant Physiol. Biochem..

[8] Gupta A., Rico-Medina A., Caño-Delgado A.I. (2020). The Physiology of Plant Responses to Drought. Science.

[9] Chaves M.M., Flexas J., Pinheiro C. (2009). Photosynthesis under Drought and Salt Stress: Regulation Mechanisms from Whole Plant to Cell. Ann. Bot..

[10] Chen G., Hu Q., Luo L., Yang T., Zhang S., Hu Y., Yu L., Xu G. (2015). Rice Potassium Transporter OsHAK1 Is Essential for Maintaining Potassium-Mediated Growth and Functions in Salt Tolerance over Low and High Potassium Concentration Ranges. Plant Cell Environ..

[11] Tardieu F., Simonneau T., Muller B. (2018). The Physiological Basis of Drought Tolerance in Crop Plants: A Scenario-Dependent Probabilistic Approach. Annu. Rev. Plant Biol..

[12] Zhang Y., Kaiser E., Zhang Y., Yang Q., Li T. (2018). Short-Term Salt Stress Strongly Affects Dynamic Photosynthesis, but Not Steady-State Photosynthesis, in Tomato (Solanum Lycopersicum). Environ. Exp. Bot..

[13] Taylaran R.D., Adachi S., Ookawa T., Usuda H., Hirasawa T. (2011). Hydraulic Conductance as Well as Nitrogen Accumulation Plays a Role in the Higher Rate of Leaf Photosynthesis of the Most Productive Variety of Rice in Japan. J. Exp. Bot..

[14] Munns R., Tester M. (2008). Mechanisms of Salinity Tolerance. Annu. Rev. Plant Biol..

[15] Liao Q., Gu S., Kang S., Du T., Tong L., Wood J.D., Ding R. (2022). Mild Water and Salt Stress Improve Water Use Efficiency by Decreasing Stomatal Conductance via Osmotic Adjustment in Field Maize. Sci. Total Environ..

[16] Astorga G.I.A., Meléndez L.A. (2010). Salinity Effects on Protein Content, Lipid Peroxidation, Pigments, and Proline in Paulownia Imperialis (Siebold & Zuccarini) and Paulownia Fortunei (Seemann & Hemsley) Grown in Vitro. Electron. J. Biotechnol..

[17] Hu Y., Schnyder H., Schmidhalter U. (2000). Carbohydrate Deposition and Partitioning in Elongating Leaves of Wheat under Saline Soil Conditions. Aust. J. Plant Physiol..

[18] Hasegawa P.M., Bressan R.A., Zhu J.K., Bohnert H.J. (2000). Plant Cellular and Molecular Responses to High Salinity. Annu. Rev. Plant Biol..

[19] Kafi M., Stewart W.S., Borland A.M. (2003). Carbohydrate and Proline Contents in Leaves, Roots, and Apices of Salt-Tolerant and Salt-Sensitive Wheat Cultivars. Russ. J. Plant Physiol..

[20] Tester M., Davenport R. (2003). Na+ Tolerance and Na+ Transport in Higher Plants. Ann. Bot..

[21] Liu X., Fan Y., Mak M., Babla M., Holford P., Wang F., Chen G., Scott G., Wang G., Shabala S. (2017). QTLs for Stomatal and Photosynthetic Traits Related to Salinity Tolerance in Barley. BMC Genom..

[22] Moula I., Boussadia O., Koubouris G., Hassine M.B., Boussetta W., van Labeke M.C., Braham M. (2020). Ecophysiological and Biochemical Aspects of Olive Tree (*Olea europaea* L.) in Response to Salt Stress and Gibberellic Acid-Induced Alleviation. S. Afr. J. Bot..

[23] Gucci R., Lombardini L., Tattini M. (1997). Analysis of Leaf Water Relations in Leaves of Two Olive (*Olea europaea*) Cultivars Differing in Tolerance to Salinity. Tree Physiol..

[24] Tattini M., Gucci R., Coradeschi M.A., Ponzio C., Everard J.D. (1995). Growth, Gas Exchange and Ion Content in *Olea europaea* Plants during Salinity Stress and Subsequent Relief. Physiol. Plant.

[25] De Lacerda C.F., Cambraia J., Oliva M.A., Ruiz H.A., Prisco J.T. (2003). Solute Accumulation and Distribution during Shoot and Leaf Development in Two Sorghum Genotypes under Salt Stress. Environ. Exp. Bot..

[26] Loreto F., Centritto M., Chartzoulakis K. (2003). Photosynthetic Limitations in Olive Cultivars with Different Sensitivity to Salt Stress. Plant Cell Environ..

[27] Delfine S., Alvino A., Villani M.C., Loreto F. (1999). Restrictions to Carbon Dioxide Conductance and Photosynthesis in Spinach Leaves Recovering from Salt Stress. Plant Physiol..

[28] Hussain S., Zhang J.H., Zhong C., Zhu L.F., Cao X.C., Yu S.M., Bohr J.A., Hu J.J., Jin Q.Y. (2017). Effects of Salt Stress on Rice Growth, Development Characteristics, and the Regulating Ways: A Review. J. Integr. Agric..

[29] Yamane K., Mitsuya S., Taniguchi M., Miyake H. (2012). Salt-Induced Chloroplast Protrusion Is the Process of Exclusion of Ribulose-1,5-Bisphosphate Carboxylase/Oxygenase from Chloroplasts into Cytoplasm in Leaves of Rice. Plant Cell Environ..

[30] Acosta-Motos J.R., Ortuño M.F., Bernal-Vicente A., Diaz-Vivancos P., Sanchez-Blanco M.J., Hernandez J.A. (2017). Plant Responses to Salt Stress: Adaptive Mechanisms. Agronomy.

[31] Chakraborty K., Bose J., Shabala L., Eyles A., Shabala S. (2016). Evaluating Relative Contribution of Osmotolerance and Tissue Tolerance Mechanisms toward Salinity Stress Tolerance in Three Brassica Species. Physiol. Plant.

[32] Araújo M., Prada J., Mariz-ponte N., Santos C., Pereira J.A., Pinto D.C.G.A., Silva A.M.S., Dias M.C. (2021). Antioxidant Adjustments of Olive Trees (*Olea europaea*) under Field Stress Conditions. Plants.

[33] Abdelaal K., Alsubeie M.S., Hafez Y., Emeran A., Moghanm F., Okasha S., Omara R., Basahi M.A., Darwish D.B.E., Ibrahim M.F.M. (2022). Physiological and Biochemical Changes in Vegetable and Field Crops under Drought, Salinity and Weeds Stresses: Control Strategies and Management. Agriculture.

[34] Tedeschini E., Proietti P., Timorato V., D’Amato R., Nasini L., Del Buono D., Businelli D., Frenguelli G. (2015). Selenium as Stressor and Antioxidant Affects Pollen Performance in *Olea Europaea*. Flora—Morphol. Distrib. Funct. Ecol. Plants.

[35] Proietti P., Nasini L., Del Buono D., D’Amato R., Tedeschini E., Businelli D. (2013). Selenium Protects Olive (*Olea europaea* L.) from Drought Stress. Sci. Hortic..

[36] Bose J., Rodrigo-Moreno A., Shabala S. (2014). ROS Homeostasis in Halophytes in the Context of Salinity Stress Tolerance. J. Exp. Bot..

[37] Elsawy H., Almalki M., Elmenshawy O., Abdel-Moneim A. (2022). In Vivo Evaluation of the Protective Effects of Arjunolic Acid against Lipopolysaccharide-Induced Septic Myocardial Injury. PeerJ.

[38] Ahmed C.B., Rouina B.B., Sensoy S., Boukhriss M., Abdullah F.B. (2009). Saline Water Irrigation Effects on Antioxidant Defense System and Proline Accumulation in Leaves and Roots of Field-Grown Olive. J. Agric. Food Chem..

[39] Bhaduri A.M., Fulekar M.H. (2012). Antioxidant Enzyme Responses of Plants to Heavy Metal Stress. Rev. Environ. Sci. Biotechnol..

[40] Keunen E., Peshev D., Vangronsveld J., Van Den Ende W., Cuypers A. (2013). Plant Sugars Are Crucial Players in the Oxidative Challenge during Abiotic Stress: Extending the Traditional Concept. Plant Cell Environ..

[41] Pang C.H., Wang B.S. (2010). Role of Ascorbate Peroxidase and Glutathione Reductase in Ascorbate-Glutathione Cycle and Stress Tolerance in Plants. Ascorbate-Glutathione Pathway and Stress Tolerance in Plants.

[42] Del Buono D., Ioli G., Nasini L., Proietti P. (2011). A Comparative Study on the Interference of Two Herbicides in Wheat and Italian Ryegrass and on Their Antioxidant Activities and Detoxification Rates. J. Agric. Food Chem..

[43] Nath M., Bhatt D., Prasad R., Gill S.S., Anjum N.A., Tuteja N. (2016). Reactive Oxygen Species Generation-Scavenging and Signaling during Plant-Arbuscular Mycorrhizal and Piriformospora Indica Interaction under Stress Condition. Front. Plant Sci..

[44] Hameed A., Goher M., Iqbal N. (2013). Drought Induced Programmed Cell Death and Associated Changes in Antioxidants, Proteases, and Lipid Peroxidation in Wheat Leaves. Biol. Plant.

[45] Boshkovski B., Doupis G., Zapolska A., Kalaitzidis C., Koubouris G. (2022). Hyperspectral Imagery Detects Water Deficit and Salinity Effects on Photosynthesis and Antioxidant Enzyme Activity of Three Greek Olive Varieties. Sustainability.

[46] Chartzoulakis K.S. (2005). Salinity and Olive: Growth, Salt Tolerance, Photosynthesis and Yield. Agric. Water Manag..

[47] Chartzoulakis K., Loupassaki M., Bertaki M., Androulakis I. (2002). Effects of NaCl Salinity on Growth, Ion Content and CO_2_ Assimilation Rate of Six Olive Cultivars. Sci. Hortic..

[48] Tattini M., Lombardini L., Gucci R. (1997). The Effect of NaCl Stress and Relief on Gas Exchange Properties of Two Olive Cultivars Differing in Tolerance to Salinity. Plant Soil.

[49] Centritto M., Loreto F., Chartzoulakis K. (2003). The Use of Low [CO_2_] to Estimate Diffusional and Non-Diffusional Limitations of Photosynthetic Capacity of Salt-Stressed Olive Saplings. Plant Cell Environ..

[50] Tabatabaei S.J. (2006). Effects of Salinity and N on the Growth, Photosynthesis and N Status of Olive (*Olea europaea* L.) Trees. Sci. Hortic..

[51] Iqbal M., Ashraf M. (2013). Gibberellic Acid Mediated Induction of Salt Tolerance in Wheat Plants: Growth, Ionic Partitioning, Photosynthesis, Yield and Hormonal Homeostasis. Environ. Exp. Bot..

[52] Tabatabaei S.J. (2007). Salinity Stress and Olive: An Overview. Plant Stress.

[53] Kchaou H., Larbi A., Chaieb M., Sagardoy R., Msallem M., Morales F. (2013). Genotypic Differentiation in the Stomatal Response to Salinity and Contrasting Photosynthetic and Photoprotection Responses in Five Olive (*Olea europaea* L.) Cultivars. Sci. Hortic..

[54] Azimi M., Khoshzaman T., Taheri M., Dadras A. (2021). Evaluation of Salinity Tolerance of Three Olive (*Olea europaea* L.) Cultivars. J. Cent. Eur. Agric..

[55] Xue F., Liu W., Cao H., Song L., Ji S., Tong L., Ding R. (2021). Stomatal Conductance of Tomato Leaves Is Regulated by Both Abscisic Acid and Leaf Water Potential under Combined Water and Salt Stress. Physiol. Plant.

[56] Li H., Hou X., Bertin N., Ding R., Du T. (2023). Quantitative Responses of Tomato Yield, Fruit Quality and Water Use Efficiency to Soil Salinity under Different Water Regimes in Northwest China. Agric. Water Manag..

[57] Syvertsen J.P., Melgar J.C., García-Sánchez F. (2010). Salinity Tolerance and Leaf Water Use Efficiency in Citrus. J. Am. Soc. Hortic. Sci..

[58] Greenway H., Munns R. (2003). Mechanisms of Salt Tolerance in Nonhalophytes. Annu. Rev. Plant Physiol..

[59] Loupassaki M.H., Chartzoulakis K.S., Digalaki N.B., Androulakis I.I. (2002). Effects of Salt Stress on Concentration of Nitrogen, Phosphorus, Potassium, Calcium, Magnesium, and Sodium in Leaves, Shoots, and Roots of Six Olive Cultivars. J. Plant Nutr..

[60] Tattini M., Bertoni P., Caselli S. (1992). Genotipic Responses of Olive Plants to Sodium Chloride. J. Plant Nutr..

[61] Tattini M. (1994). Ionic Relations of Aeroponically-Grown Olive Genotypes, during Salt Stress. Plant Soil.

[62] Marschner H. (1955). Mineral Nutrition of Higher Plants.

[63] Zhu J.K. (2001). Plant Salt Tolerance. Trends Plant Sci..

[64] Alkahtani S., Alarifi S., Alkahtane A.A., Albasher G., AL-Zharani M., Alhoshani N.M., AL-Johani N.S., Aljarba N.H., Saquib Hasnain M. (2021). Pyrroloquinoline Quinone Alleviates Oxidative Damage Induced by High Glucose in HepG2 Cells. Saudi J. Biol. Sci..

[65] Meloni D.A., Oliva M.A., Martinez C.A., Cambraia J. (2003). Photosynthesis and Activity of Superoxide Dismutase, Peroxidase and Glutathione Reductase in Cotton under Salt Stress. Environ. Exp. Bot..

[66] Munns R. (1993). Physiological Processes Limiting Plant Growth in Saline Soils: Some Dogmas and Hypotheses. Plant Cell Environ..

[67] Mittler R. (2017). ROS Are Good. Trends Plant Sci..

[68] Sofo A., Dichio B., Xiloyannis C., Masia A. (2005). Antioxidant Defences in Olive Trees during Drought Stress: Changes in Activity of Some Antioxidant Enzymes. Funct. Plant Biol..

[69] Jiao L., Wang L., Zhou Q., Huang X. (2017). Stomatal and Non-Stomatal Factors Regulated the Photosynthesis of Soybean Seedlings in the Present of Exogenous Bisphenol A. Ecotoxicol. Environ. Saf..

[70] Yang Z., Li J.L., Liu L.N., Xie Q., Sui N. (2019). Photosynthetic Regulation Under Salt Stress and Salt-Tolerance Mechanism of Sweet Sorghum. Front. Plant Sci..

[71] Chazen O., Hartung W., Neumann P.M. (1995). The Different Effects of PEG 6000 and NaCI on Leaf Development Are Associated with Differential Inhibition of Root Water Transport. Plant Cell Environ..

[72] Martin-Prevel P., Martin-Prevel P., Gagnard J., Gautier P. (1984). L’analyse Végétale Dans Le Contrôle de l’alimentation Des. Plantes Tempérées et Tropicales.

[73] Teranishi Y., Tanaka A., Osumi M., Fukui S. (1974). Catalase Activities of Hydrocarbon-Utilizing Candida Yeasts. Agric. Biol. Chem..

[74] Nakano Y., Asada K. (1981). Hydrogen Peroxide Is Scavenged by Ascorbate-Specific Peroxidase in Spinach Chloroplasts. Plant Cell Physiol..

[75] Pütter J. (1974). Peroxidases. Methods Enzym. Anal..

[76] Duncan D.B. (1955). Multiple Range and Multiple F Tests. Biometrics.

